# Resistance to genetic insect control: Modelling the effects of space

**DOI:** 10.1016/j.jtbi.2016.10.014

**Published:** 2017-01-21

**Authors:** Benjamin Watkinson-Powell, Nina Alphey

**Affiliations:** a Department of Life Sciences, Imperial College London, Silwood Park Campus, Buckhurst Road, Ascot, Berkshire SL5 7PY, United Kingdom; b Mathematical Ecology Research Group, Department of Zoology, University of Oxford, South Parks Road, Oxford OX1 3PS, United Kingdom

**Keywords:** Pest insects, Self-limiting RIDL, Sterile insect technique (SIT), Mathematical modelling, Spatial dynamics

## Abstract

Genetic insect control, such as self-limiting RIDL^2^ (Release of Insects Carrying a Dominant Lethal) technology, is a development of the sterile insect technique which is proposed to suppress wild populations of a number of major agricultural and public health insect pests. This is achieved by mass rearing and releasing male insects that are homozygous for a repressible dominant lethal genetic construct, which causes death in progeny when inherited. The released genetically engineered (‘GE’) insects compete for mates with wild individuals, resulting in population suppression. A previous study modelled the evolution of a hypothetical resistance to the lethal construct using a frequency-dependent population genetic and population dynamic approach. This found that proliferation of resistance is possible but can be diluted by the introgression of susceptible alleles from the released homozygous-susceptible GE males. We develop this approach within a spatial context by modelling the spread of a lethal construct and resistance trait, and the effect on population control, in a two deme metapopulation, with GE release in one deme. Results show that spatial effects can drive an increased or decreased evolution of resistance in both the target and non-target demes, depending on the effectiveness and associated costs of the resistant trait, and on the rate of dispersal. A recurrent theme is the potential for the non-target deme to act as a source of resistant or susceptible alleles for the target deme through dispersal. This can in turn have a major impact on the effectiveness of insect population control.

## Highlights

• Spatial model of releasing ‘genetically sterile’ males into target population.
• Resistant allele *R* reduces lethality of genetic construct, susceptible allele *S*.
• Non-target deme can act as source of *S* alleles, diluting resistance in target deme.
• Non-target deme can act as source of *R* alleles, exacerbating spread in target deme.
• Insect population control outcomes depend on resistance traits and dispersal rates.

## Graphical abstract

**Figure:**
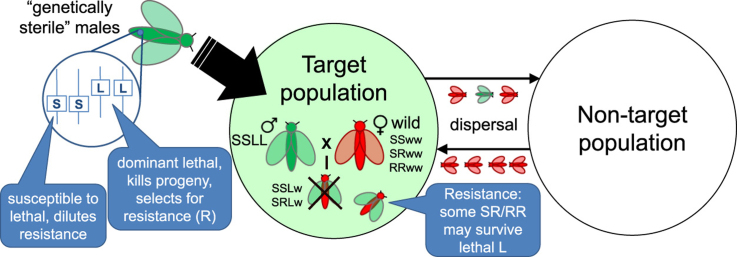

## 1 Introduction

The control of insect pests is a major area of concern for both public health and agriculture, as these pests cause widespread harm and economic damage. Diseases such as malaria and dengue fever, transmitted by *Anopheles* and *Aedes* mosquitoes respectively, are particularly damaging, with the former being responsible for around 584,000 deaths in 2013 (World Health Organisation, 2014), whilst insect pests are responsible for a large proportion of the 18% annual crop production losses attributable to animal pests overall (Oerke, 2006).

The sterile insect technique (SIT) is a method that has been used since the 1950s to control pest populations and involves releasing large numbers of mass reared insects into natural populations (Knipling, 1955). These insects, having been sterilised by irradiation, compete for mates with wild insects but do not produce any viable offspring, thus reducing the total number of offspring contributing to the next generation. If the number of insects released is large enough and released over a long enough period this can lead to local population suppression or elimination. The technique has been used to eradicate pests such as the screwworm fly *Cochliomyia hominivorax* in the USA and Mexico, and the Mediterranean fruit fly (‘Medfly’) *Ceratitis capitata* from various locations in the Americas (reviewed by Alphey et al., 2010).

A self-limiting genetic technology, referred to in published literature as ${\mathrm{RIDL}}^{®}$ (Release of Insects carrying a Dominant Lethal), is a development of the sterile insect technique that involves the release of genetically engineered (‘GE’) insects homozygous for a dominant lethal genetic construct instead of being irradiated (Alphey, 2014, Thomas et al., 2000). These insects compete for mates with wild type insects and pass the construct onto their offspring, causing them to die before they fully mature (supplementary figure S1). The timing of death during insect development can be engineered in order to maximise population suppression. For example, early acting lethality is preferable for some agricultural pests such as Medfly where the larval stages cause the most damage (Schetelig et al., 2007). Late acting lethality on the other hand is used for insects, such as mosquitoes, that have a density dependent mortality phase during their development; if the lethality acts after this phase, population suppression is maximised (Atkinson et al., 2007, Phuc et al., 2007).

The efficiency of both the traditional sterile insect technique and its genetic variants are greatly increased if only males are released, as these individuals must then disperse and find wild-type females to mate with (Rendon et al., 2004). The separation of males and females can be achieved mechanically on a large enough scale for a limited number of species including screwworm and *Aedes aegypti* mosquitoes, and using classical genetics for Medfly, but is not 100% effective (Alphey et al., 2010). The need for male only release is particularly pertinent for insects such as mosquitoes where only females bite and transmit diseases.

The evolution of resistance to both chemical insecticides and insecticidal (Bt toxin) crops is already severely impeding population control efforts in a number of species (Nicholson, 2007, Tabashnik et al., 2009) and has the potential to impact severely on the efficacy of the sterile insect technique. Behavioural resistance to conventional sterile insect technique has already been observed where selection favours wild-type females that alter their mating preferences to avoid lab reared males (McInnis et al., 1996). Although none has yet been detected in lab reared or sampled wild insect populations, there exists the distinct possibility that a genetic resistance to the lethal construct may emerge (Alphey et al., 2011, Gong et al., 2005).

The emergence of resistance to an engineered lethal genetic construct could occur through selection for an existing allele that may be currently undetected due to low frequency, or through mutation in the genes affecting susceptibility to the construct (Alphey et al., 2011). In either case, a strong advantage would be conferred once the lethal construct was introduced into a population. However, unless it conferred some pleiotropic advantage, a resistance trait would not be under any positive selective pressure in the absence of the lethal construct, and so would likely only be present at a very low frequency in wild populations. For comparison, naturally occurring insect resistance to Bt toxins without widespread prior exposure has been found at frequencies of around 10^−3^ (Mahon et al., 2007). As no resistance to a genetic lethal construct has been detected, we must consider its potential key properties. Hypothetical resistance could provide either complete or partial protection from the lethal effects. Such a resistance trait could impose costs on the insect, particularly if it acts by simply increasing metabolism to counter the lethal mechanism.

In a previous study (Alphey et al., 2011) we used a combined population genetic and population dynamic model to investigate potential scenarios for the spread of both the lethal construct and a pre-existing hypothetical physiological resistance allele. The effect of varying the effectiveness and cost of resistance, along with the size of the GE release, on allele frequencies and population size was tested for varying levels of dominance in the resistance gene. We found that the evolution of the resistant allele frequency was driven by selection pressure from the lethal effects of the construct as expected. However, the spread of the resistance allele was limited by resistance dilution from the influx of susceptible alleles from the released modified males, which were homozygous susceptible at the resistant locus. This dilution effect could prevent the emergence of, or even reverse the spread of resistance and enable effective population suppression. The model looked at both bisex–lethal GE strains, where all progeny (of both sexes) are targeted by the construct, and female-lethal strains, which target females but leave males unaffected (Heinrich and Scott, 2000, Thomas et al., 2000). These were assumed to be fully dominant and 100% lethal to the target sex, as is usually the case with lab tested strains that are selected for further development (Ant et al., 2012, Fu et al., 2007, Gong et al., 2005, Jin et al., 2013, Phuc et al., 2007). The overall effectiveness of GE control was measured by simulating population size over time, as the number of individuals emerging in each generation is an effective indicator of economic or public health damage.

That model (Alphey et al., 2011) took no account of the spatial distribution of the target and released populations. We know, however, that spatial structure has the potential to be a major factor determining the efficacy of GE-SIT and other genetic control methods. Yakob et al. (2008) used a network approach to model the effect of spatial population clustering on the efficacy of GE-SIT control, finding that more highly clustered insect populations were easier to suppress. This was because once a local cluster was eliminated it could not easily be repopulated due to its isolation (Yakob et al., 2008). Another genetic insect control method, using a synthetic homing endonuclease gene (HEG), was modelled by North et al. (2013) in an individual based simulation, revealing that control could fail if mosquito resources, and therefore local mosquito populations, were too isolated. The size of release sites has also been found to impact upon the effectiveness of a genetic or radiation-based sterile insect technique control. Seirin Lee et al. (2013) modelled a barrier control method, whereby insects are released in order to halt the invasion of a wild-type wave, finding that the size of the optimal release region depended on the dispersal rate of the invading population.

Here we explore the dynamics of a genetic lethal construct and a corresponding resistance allele in a spatial model. Specifically, we address the question of how a population targeted with the application of a treatment (GE release) interacts with a second non-target population, which is linked to the target population through dispersal. A simple two deme metapopulation model is sufficient for this investigation of how an asymmetrical interaction between target and non-target populations affects the evolution of resistance. The opposing forces of selection for, and dilution of, resistance highlighted in the original non-spatial model could play out in a different manner when space is taken into account. We hypothesise that gene flow of the GE construct into surrounding areas, outside the target population, and immigration of individuals from surrounding areas into the target population, could alter the frequency evolution of the resistance gene and consequently affect the efficacy of genetic control of the target population. We investigate the influence of resistance traits and dispersal rates on predicted outcomes for a bisex–lethal genetic system.

## 2 Methods

The model used in this study is based on that previously published (Alphey et al., 2011). It is a discrete-generation frequency dependent population genetic and population dynamic model, with random mating, no mutation, and a 1:1 sex ratio. The original model was of a closed homogeneous population with no immigration or emigration. The underlying mathematical processes described in Sections 2.1 and 2.2 are essentially the same as those in the original model, while the spatial dynamics described in Section 2.3 are new, extending the investigation to explore the effects of spatial heterogeneity. A summary of the parameters and variables used in this model can be found in Table 1.

### 2.1 Population genetics

Within the insect population there are two genetic loci being modelled, the insertion or absence of the lethal construct, and the locus of resistance. The specific genotype of insects will affect their relative fitness and survival.

The gene affecting the susceptibility of an individual to the lethal genetic construct is assumed to have a single autosomal locus. The two alternative alleles for this gene are resistant *R* (at frequency *p* in the current adult generation) and susceptible *S* (at frequency q=1−p in the current adult generation), meaning that there are three possible genotypes at this locus (*SS*, *SR* and *RR*). In all simulations presented here the initial frequency of the *R* allele in the population is $p_{0}=0.001$, as this represents the very low frequency that might be expected of a recent mutation or a pre-existing allele that is not readily detectable (Alphey et al., 2011).

The level of protection that a resistant genotype provides to an individual is governed by the parameter governing susceptibility to the lethal construct *γ*_*i*_ where *i* is the resistant/susceptible genotype. Susceptible homozygotes have no protection from the effects of the lethal construct ($\gamma_{\mathrm{SS}}=1$), while homozygote resistant individuals have reduced susceptibility to the construct ($\gamma_{\mathrm{RR}}<\gamma_{\mathrm{SS}}$). Resistance can be complete ($\gamma_{\mathrm{RR}}=0$), providing complete protection from the effects of the lethal construct, or incomplete ($1>\gamma_{\mathrm{RR}}>0$) with different levels of dominance affecting the susceptibility of the heterozygote (*SR*) genotype. In this paper a number of different terms are used to describe the different types of potential resistance studied: dominant complete ($\gamma_{\mathrm{SR}}=\gamma_{\mathrm{RR}}=0$), partially dominant complete ($1>\gamma_{\mathrm{SR}}>0$, $\gamma_{\mathrm{RR}}=0$), co-dominant complete ($\gamma_{\mathrm{SR}}=0.5$, $\gamma_{\mathrm{RR}}=0$), and partially-dominant incomplete ($1>\gamma_{\mathrm{SR}}>\gamma_{\mathrm{RR}}>0$) (summarised in Table 2).

The resistant allele may or may not have associated fitness costs, depending on its mechanism, which are represented here by the fitness of genotype *i* (*ψ*_*i*_) relative to the fitness of homozygous susceptible wild-type insects ($\psi_{\mathrm{SS}}=1$). In this study four different types of resistance were tested: no costs ($\psi_{\mathrm{SR}}=\psi_{\mathrm{RR}}=1$), ‘minor costs’ ($\psi_{\mathrm{SR}}=0.95$, $\psi_{\mathrm{RR}}=0.85$), ‘fit resistance’ ($\psi_{\mathrm{SR}}=0.9$, $\psi_{\mathrm{RR}}=0.7$), and ‘costly resistance’ ($\psi_{\mathrm{SR}}=0.2$, $\psi_{\mathrm{RR}}=0.1$) (summarised in Table 2). These parameter values were chosen to aid comparison with Alphey et al. (2011) where they were found to be effective for determining the effect of the magnitude of resistance costs.

The lethal genetic construct itself also has a single autosomal locus with the assumption that there is no linkage between this and the locus controlling resistance to the construct. The two possible alleles at this locus are the dominant *L* (at frequency *l* in the current adult generation), where the construct has been inserted, and the wild-type *w* (at frequency 1−l in the current adult generation), where the construct is absent, giving three possible genotypes (*ww*, *Lw* and *LL*). Where the construct is present it imposes a relative fitness cost *ϵ*_*k*_, where *k* indicates either the target or non-target sex. In this study a fully bisex–lethal construct is investigated so a maximum fitness penalty is always applied to both sexes (*ϵ*_*k*_=1). The relative fitness of genotype *j* (*ww*, *Lw* or *LL*) is given by:

(1) $$\Omega_{j}={\left(1-\epsilon_{k}\right)}^{\eta_{j}}$$

where *η*_*j*_ is the number of *L* allele copies present (0=*ww*, 1=*Lw*, 2=*LL*). As the construct is bisex–lethal and dominant $\Omega_{\mathrm{ww}}=1$ and $\Omega_{\mathrm{Lw}}=\Omega_{\mathrm{LL}}=0$. When susceptibility to the construct (*γ*_*i*_) is included in the equation it acts as a scaling factor on the fitness (survival) penalty *ϵ*_*k*_, while the cost of resistance (*ψ*_*i*_) modifies the resulting fitness equation to give:

(2) $$\Omega_{\mathrm{ijk}}=\psi_{i}{\left(1-\epsilon_{k}\gamma_{i}\right)}^{\eta_{j}}$$

The three possible genotypes given by *i* (*SS*, *SR* and *RR*), the three by *j* (*ww*, *Lw* and *LL*) and the two sexes means that there are potentially a total of 18 genotypes used in this model. In practice because only a bisex–lethal construct is modelled, and we assume an equal sex ratio, there are essentially only 9 genotypes as the male and female genotypes always have equal frequencies.

The genetic control being used on the wild insect population is modelled by the addition of adult males that are homozygous for both the susceptible allele and the lethal construct (*SSLL*) at a fixed ratio *d* to the total number of males in the wild population at that generation. While this proportional release policy may be difficult to implement in practice, it was included in the original model as it allowed the change in allele frequencies to be calculated independently of population size. In a spatial model where dispersal is dependent on population size this rationale is no longer strictly true, however, the assumption still serves to simplify the calculations involved in the model and allows direct comparison with previously published results. The addition of GE males occurs as the current generation in the wild population reaches maturity, prior to mating. Wild and released insects are assumed to mix homogeneously in the target population and random mating subsequently occurs between females and all male genotypes.

The frequencies of each zygote genotype are calculated post-mating after which Eq. (2) is applied to calculate their relative fitness and survival. In this way the effects of both the lethal construct and the costs of resistance act during the larval stage of the insects' life cycle. Those insects that survive then mature to become the adults of the next generation. The original non-spatial model used a simulation approach to calculate the changes in allele and genotype frequencies due to the large number of genotypes which would otherwise result in a complex system of difference equations that cannot be readily solved analytically. Adding a spatial element further complicates the model, so a simulation approach is even more essential. Simulations were performed in R (R Core Team, 2016).

### 2.2 Population dynamics

The insect population grows each generation at a rate determined by the basic reproductive rate, *R*_0_, which is the average number of offspring produced per adult pest insect in its lifetime (a single discrete generation). Where separate female population size is recorded, as it is here, and with an equal sex ratio this can be expressed as the number of female offspring produced per adult female. For the generic agricultural and public health pests (target species suitable for SIT) that are simulated in this model, an *R*_0_ value of 7.5 is used; a plausible *R*_0_ value for *A. aegypti* mosquitoes for example would be in the range 3–11 (Dye, 1984). In a density-independent population, with the assumption that half of the adults are female, growth is calculated by:

(3) $$N_{t+1}=2R_{0}F_{t}\sigma_{t}$$

(4) $$F_{t+1}=R_{0}F_{t}\sigma_{t}^{\left(F\right)}$$

where *N*_*t*_ is the adult pest population size at generation *t* relative to the initial population size ($N_{0}=1$). 2R_0_ here is the average number of progeny (male and female) produced per adult female. *σ*_*t*_ and $\sigma_{t}^{\left(F\right)}$ are the proportion of all offspring and of female offspring respectively that survive to adulthood (calculated using the fitness values calculated from Eq. (2)). In a bisex–lethal model these values are the same as there are no differential survival rates between the sexes.

All the simulations presented in this study feature density dependent population dynamics in order to represent a realistic field setting. This model adapts an equation from Bellows (1981):

(5) $${\overset{˜}{N}}_{t+1}=R_{0}{\overset{˜}{N}}_{t}e^{-\alpha {\overset{˜}{N}}_{t}}$$

where $\overset{˜}{N}$ is the absolute population size, *α* is the strength of the density dependence, and 1/α is related to the carrying capacity of the habitat. Because our model uses relative population size rather than the absolute, a substitution of variables is made where population sizes are measured relative to their initial equilibrium value ($N_{t}={\overset{˜}{N}}_{t}/{\overset{˜}{N}}^{*}$ and $F_{t}={\overset{˜}{F}}_{t}/{\overset{˜}{F}}^{*}$), so that ($N_{0}=N^{*}=1$ and $F_{0}=F^{*}=0.5$). The non-zero equilibrium for absolute population size from Eq. (5) is:

(6) $${\overset{˜}{N}}^{*}=\frac{\log(R_{0})}{\alpha }$$

which can be substituted into Eq. (5) giving:

(7) $${\overset{˜}{N}}_{t+1}=R_{0}{\overset{˜}{N}}_{t}R_{0}^{-N_{t}}$$

As the density dependent term now uses the relative population size *N*_*t*_ it can now be applied to the density-independent equations (3), (4) to give the population growth used in this model:

(8) $$N_{t+1}=2R_{0}F_{t}R_{0}^{-N_{t}}\sigma_{t}$$

(9) $$F_{t+1}=R_{0}F_{t}R_{0}^{-N_{t}}\sigma_{t}^{\left(F\right)}$$

Density dependent mortality is assumed to act before the lethal construct and costs of resistance, so the relative survival terms (*σ*) are applied to the density dependence formula.

### 2.3 Spatial modelling

In order to explore spatial dynamics this model uses a two deme metapopulation structure with GE release in only one ‘target’ deme and dispersal between this and the other ‘non-target’ deme (supplementary figure S2). Both demes are of equal size and with an equal and constant rate of dispersal *m* in each direction (i.e. *m*=0.1 represents 10% of individuals migrating). The system is otherwise closed with no immigration or emigration beyond the two demes.

Dispersal occurs after mating but before females have laid their eggs (and therefore before the larval phase density dependence and survival calculations). This means that although both sexes could disperse in reality, only female dispersal is calculated here as at this point only they can contribute to the next generation of insects. Conceptually, mated females may be thought of as containers for fertilised eggs, which either transport those eggs/zygotes to another deme or remain in the deme where mating occurred. All 18 zygote genotype frequencies, along with the adult female population size, must be adjusted every generation to account for dispersal:

(10) $$Z_{t+1}^{\left(T\right)}=(1-m)Z_{t}^{\left(T\right)}F_{t}^{\left(T\right)}+{\mathrm{mZ}}_{t}^{\left(\mathrm{NT}\right)}F_{t}^{\left(\mathrm{NT}\right)}$$

(11) $$Z_{t+1}^{\left(\mathrm{NT}\right)}=(1-m)Z_{t}^{\left(\mathrm{NT}\right)}F_{t}^{\left(\mathrm{NT}\right)}+{\mathrm{mZ}}_{t}^{\left(T\right)}F_{t}^{\left(T\right)}$$

where *Z* is a matrix of genotype frequencies, (T) indicates a variable belonging to the target deme, and (NT) a variable belonging to the non-target deme. The resulting genotype frequencies are then rescaled so that $Z_{t+1}$ sums to 1. The female population size in this previous generation must also be adjusted to take into account the migration of adult females:

(12) F^t(T)=(1−m)Ft(T)+mFt(NT)

(13) F^t(NT)=(1−m)Ft(NT)+mFt(T)

After dispersal is complete the zygote genotype frequencies in each deme comprise the next generation and are adjusted by their individual survival rates. The (adult) population size of that next generation is then calculated, with the migration adjusted female population size (F^t) from the previous generation, using Eqs. (8), (9). In these equations Nt=2Ft^ (in the density dependence term) as density dependent larval mortality is a function of adult female numbers from the previous generation. This process simulates the eggs being laid, the larvae emerging, and their maturation to adulthood.

When dispersal is removed from this model (*m*=0) the target deme functions as a version of the original non-spatial model with bisex–lethal GE release, while the non-target deme functions as a version with no release of GE insects. In this way the spatial model was validated by confirming that results were consistent with results from the underlying non-spatial model. The insect life cycle simulated in this model is summarised in supplementary figure S3.

### 2.4 Simulations

In all simulations the populations were assumed to be naive to the GE construct and did not contain any *L* alleles ($l_{0}=0$). The initially present genotypes are in Hardy–Weinberg equilibrium ($\mathrm{SSww}=q_{0}^{2}$, $\mathrm{SRww}=2p_{0}q_{0}$, $\mathrm{RRww}=p_{0}^{2}$) while the other genotypes involving the *L* allele only arise after GE insects have been released and mate with wild females.

To illustrate the range of complex dynamics that can arise in a spatially explicit model, a number of qualitatively and quantitatively different resistance costs and susceptibilities (summarised in Section 2.1) were chosen and run in different combinations. Also in order to simplify results a release ratio of *d*=20, a realistic ratio that might be used in a real control programme, was used in most simulations except for in some early tests (Alphey et al., 2011). All simulations were run for the number of generations required for equilibrium values to be reached, or if this took a very long period of time, until the qualitative pattern of results became clear. As the primary purpose of this study was to investigate spatial dynamics, all simulations were run at three dispersal rates (m=0.01,0.05,0.1) with additional rates being tested if this was insufficient to determine the effect of the parameter. Time series of both the *L* and *R* allele frequencies along with the relative population size *N* at every generation were recorded for both demes in every simulation run.

In the first phase of simulation modelling a non-spatial versus spatial comparison was made using a strong partially dominant incomplete resistance trait ($\gamma_{\mathrm{SR}}=0.2$, $\gamma_{\mathrm{RR}}=0.1$) with no associated costs ($\psi_{\mathrm{SR}}=\psi_{\mathrm{RR}}=1$). This initial spatial simulation was also run with a low (*d*=1) and very high (*d*=50) release ratio to determine whether this parameter was capable of producing qualitatively different or unexpected results. Following this, the same simulation (with *d*=20) was run with each of the three costs of resistance detailed in Section 2.1 (minor costs, fit resistance and costly resistance). The role of various susceptibilities of resistance was then investigated under a variety of these cost regimes (see Table 2 for resistance effects and costs).

In all simulations, we consider an equilibrium to have been reached if a value, or the periodic limit of an oscillating value, converges to within 5 decimal places. Furthermore, due to the deterministic nature of the model, using continuous state variables, the total fixation (or loss) of an allele is impossible and values instead asymptote at these points (referred to as near fixation).

## 3 Results

### 3.1 Spatial vs. non-spatial for a no-cost resistance

**Fig. 1:**
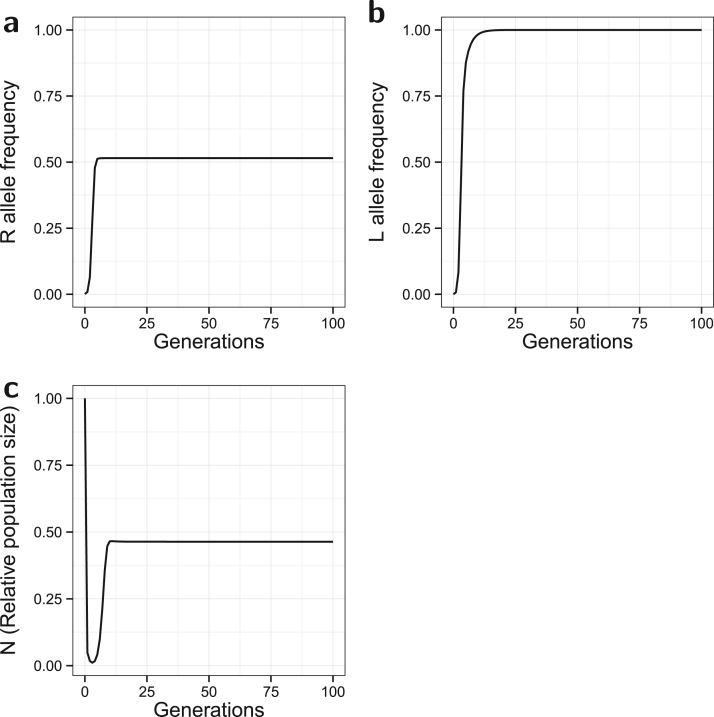
*Non-spatial model*. Evolution of the *R* allele frequency (a), the *L* allele frequency (b), and the change in the relative population size over time (c). The model has a release ratio of *d*=20, and a strong partially dominant incomplete resistance ($\gamma_{\mathrm{SR}}=0.2$, $\gamma_{\mathrm{RR}}=0.1$) with no associated costs ($\psi_{\mathrm{SR}}=\psi_{\mathrm{RR}}=1$). The spread of resistance leads to fixation of the lethal construct, and reduces the effectiveness of population control.

In the original non-spatial model, a strong, partially dominant, incomplete resistant (*R*) allele with no fitness costs quickly spreads to reach an equilibrium of p≈0.515 in 13 generations (Fig. 1). This allows the lethal construct (*L*) allele to spread through the population, due to the increased survival of *L*-bearing offspring, and reach near fixation ($l^{*}\approx 1.00$) in 31 generations. The *R* allele is prevented from reaching fixation due to continuing dilution by the released *SSLL* males. The relative population size (*N*) initially falls rapidly from 1.00 to a low of 0.011 in three generations before recovering, at around the time that *p* increases above 0.5 (*R* is more common than *S*), to an equilibrium of $N^{*}\approx 0.464$ by generation 25. Therefore population suppression is still achieved, albeit to a lesser extent. With no resistance the population would be eliminated ($N^{*}\approx 0$ , to 5 d.p.) in 10 generations.

**Fig. 2:**
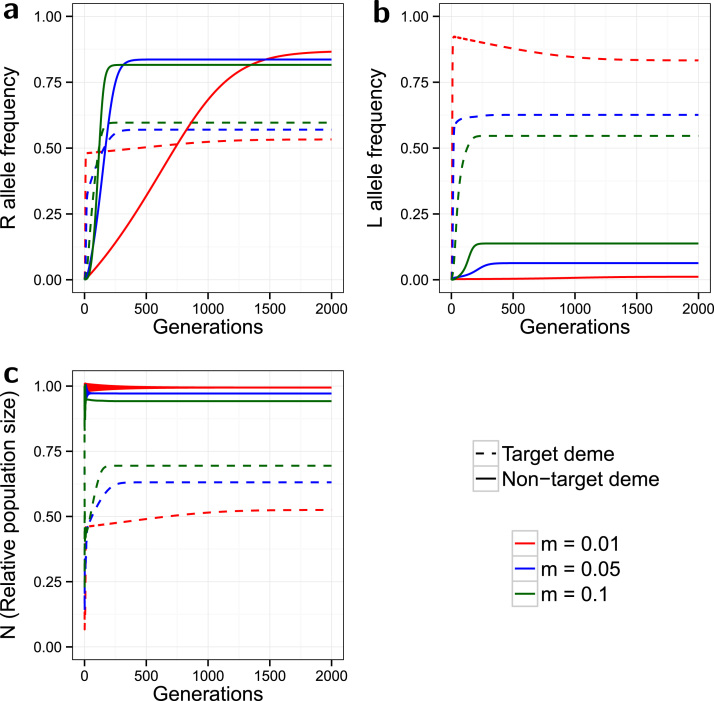
*Spatial model, no-cost resistance*. Evolution of the *R* allele frequency (a), the *L* allele frequency (b), and the change in the relative population size over time (c). The model is spatial, with release ratio *d*=20, and a strong partially dominant incomplete resistance ($\gamma_{\mathrm{SR}}=0.2$, $\gamma_{\mathrm{RR}}=0.1$) with no associated costs ($\psi_{\mathrm{SR}}=\psi_{\mathrm{RR}}=1$). Dashed lines indicate the target deme, solid lines indicate the non-target deme, and the line colours indicate the simulated dispersal rate (see legend). 2000 of the 3000 simulated generations are shown. The non-target deme acts as a source of *R* alleles, which in turn reduces the effectiveness of control in the target deme. Higher dispersal increases the magnitude of this impact.

In the two deme spatial model, the system takes far more generations to reach equilibrium, (Fig. 2). For all values of the dispersal rate *m* tested (0.01, 0.05 and 0.1) the *L* allele does not reach fixation in either deme, but reaches a much higher equilibrium frequency in the target deme compared to the non-target deme. In the target deme $l^{*}$ is lower with higher values of *m*, whereas in the non-target deme it is higher with higher dispersal; the resultant effect is that the target and non-target equilibrium frequencies are closer together for higher values of *m*. This is due to higher rates of dispersal shifting more *L* alleles from the target to the non-target deme. Instead of increasing to an equilibrium in a sigmoidal curve, the *L* allele frequency with low dispersal (*m*=0.01) increases to a local peak before falling gradually to equilibrium.

Strikingly, the *R* allele reaches a markedly higher equilibrium frequency in the non-target compared to the target deme. This is due to strong positive selection for the no-cost resistance, even with a low *L* allele frequency, combined with only weak dilution from the progeny of released *SSLL* males. This resistance dilution in the non-target deme will be even weaker with lower dispersal rates. As with the *L* allele, higher *m* values produce closer equilibria between the two demes, with the effect of increasing *p* in the target and decreasing *p* in the non-target deme. The rapid spread of resistance in the non-target deme causes it to act as a source of *R* alleles for the target deme; this effect increases with greater dispersal.

Significant population suppression is most clearly observed in the target deme where, as in the non-spatial model, *N* initially drops very rapidly then climbs back to an equilibrium between 0.5 and 0.75. Target population suppression is inferior to that of the non-spatial model (Fig. 1). This is partly due to the net migration of insects from the non-target deme, which retains a higher population size, but it is also due to the slightly higher frequency of resistance in the target deme (compared to Fig. 1). In contrast, the non-target deme shows little population suppression due to a relatively low influx of *L* alleles.

With higher values of *m* the target $N^{*}$ is higher (inferior population suppression) but is lower in the non-target deme. For the target deme this is due to a combination of the lower *L* equilibrium, the higher *R* equilibrium, and a greater general loss of insects migrating to the non-target deme. Again, the two demes’ equilibria are closer in value at higher dispersal rates. Simulations conducted with alternative release ratios (*d*) produced no qualitatively novel results beyond those we have already described (Alphey et al., 2011) (Supplementary Material Section 3.1 and figure S4).

### 3.2 Adding costs of resistance

**Fig. 3:**
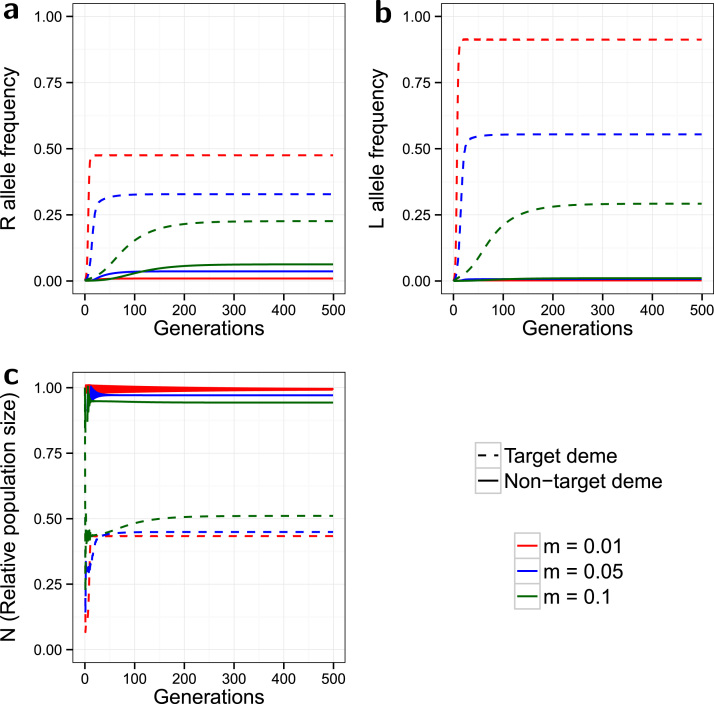
*Minor costs of resistance*. Evolution of the *R* allele frequency (a), the *L* allele frequency (b), and the change in the relative population size over time (c). The model is spatial, with release ratio *d*=20, and a strong partially dominant incomplete resistance ($\gamma_{\mathrm{SR}}=0.2$, $\gamma_{\mathrm{RR}}=0.1$) with minor costs ($\psi_{\mathrm{SR}}=0.95$, $\psi_{\mathrm{RR}}=0.85$). Dashed lines indicate the target deme, solid lines indicate the non-target deme, and the line colours indicate the simulated dispersal rate (see legend). The non-target deme acts as a source of *S* alleles, thereby enhancing the effectiveness of target population control.

Running the same simulations with minor costs of resistance results in very different patterns of equilibrium frequencies (Fig. 3) compared to the no-cost model. In contrast to the no-cost model (compare Fig. 2, Fig. 3), the *R* equilibrium is higher in the target deme than in the non-target, while all $p^{*}$ values are lower than in any of the no-cost simulations. The costs of resistance lower the selective pressure for the *R* allele by reducing the number of *SR* and *RR* individuals surviving to maturity, particularly in the non-target deme which has lower exposure to the *L* allele. Increasing *m* still results in deme equilibria being closer in value, due to greater mixing between the populations. However as the target $p^{*}$ is higher than in the non-target deme, increased dispersal now has the opposite effect on the equilibrium of each individual deme (increasing *m* reduces and increases $p^{*}$ in the target and non-target demes respectively). The non-target deme now becomes a source of *S* alleles for the target deme. The *L* allele spreads in the target deme, and to a lower equilibrium with higher *m* as in the no-cost model. However *L* barely spreads in the non-target deme with any value of *m* because of the low number of resistant individuals. Due to the lower overall resistance spread, target population suppression is greater for all values of *m* compared to the no-cost model, and is again superior with a lower *m*. Equilibrium frequencies for non-target *N* are not markedly different from those in the no-cost model.

**Fig. 4:**
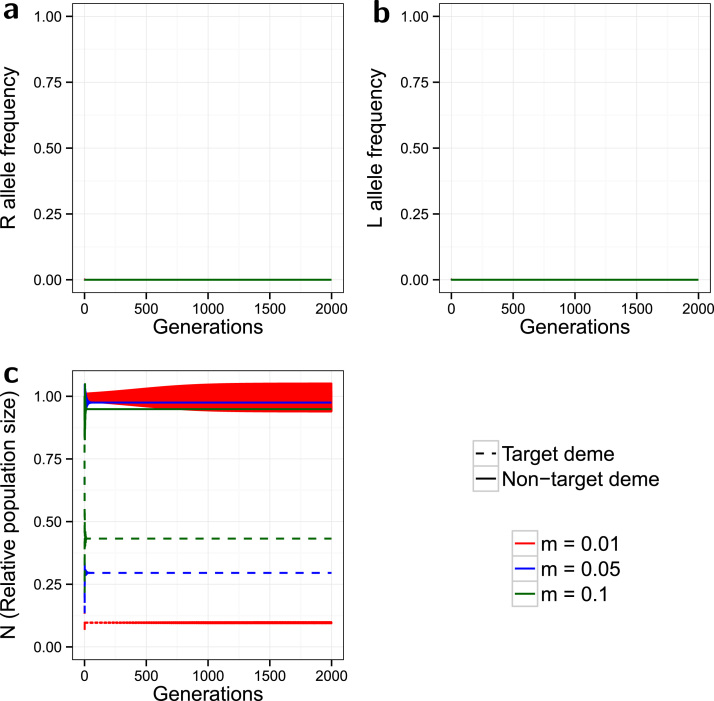
*Greater costs of resistance*. Evolution of the *R* allele frequency (a), the *L* allele frequency (b), and the change in the relative population size over time (c). The model is spatial, with release ratio *d*=20, a strong partially dominant incomplete resistance ($\gamma_{\mathrm{SR}}=0.2$, $\gamma_{\mathrm{RR}}=0.1$), and costly resistance ($\psi_{\mathrm{SR}}=0.2$, $\psi_{\mathrm{RR}}=0.1$). Dashed lines indicate the target deme, solid lines indicate the non-target deme, and the line colours indicate the simulated dispersal rate (see legend). Note the longer time scale (2000 generations) than shown in earlier figures. The high-cost resistance goes extinct in both demes; control of the target population is only tempered by immigration from the non-target deme.

Higher resistance costs result in a reduced spread of both the resistant allele and the lethal construct in both demes (Supplementary Material Section 3.2 and figure S5 for intermediate cost ‘fit resistance’). In simulations with costly resistance (Fig. 4) neither the *R* nor *L* alleles spread in either deme, while target population suppression is superior to that with any of the other costs of resistance tested. A new behaviour seen in this set of results, is that of the oscillations in the non-target *N* with *m*=0.01, which are much larger than previously seen and do not converge, instead reaching a stable period 2 cycle (with limits 0.939 and 1.051 converging to 5 d.p.).

We have shown a variety of outcomes that depend on the fitness costs of the resistant allele, which we have assumed throughout this section to be strong partially dominant and incomplete. If there are no fitness costs the *R* allele spreads through the non-target deme which becomes a source of *R* alleles and negatively affects the suppression of the target population (Fig. 2). Minor fitness costs prevent the *R* allele from spreading in the non-target deme, which acts as a source of *S* alleles and thereby dilutes the resistance in the target deme (Fig. 3). Resistance is unable to spread in the target population anyway if its fitness costs are very high (Fig. 4). To explore the nature of these shifts in behaviour, we examine plots of equilibria (rather than time dynamics) against the full spectrum of fitness costs in resistant heterozygotes and in homozygotes.

**Fig. 5:**
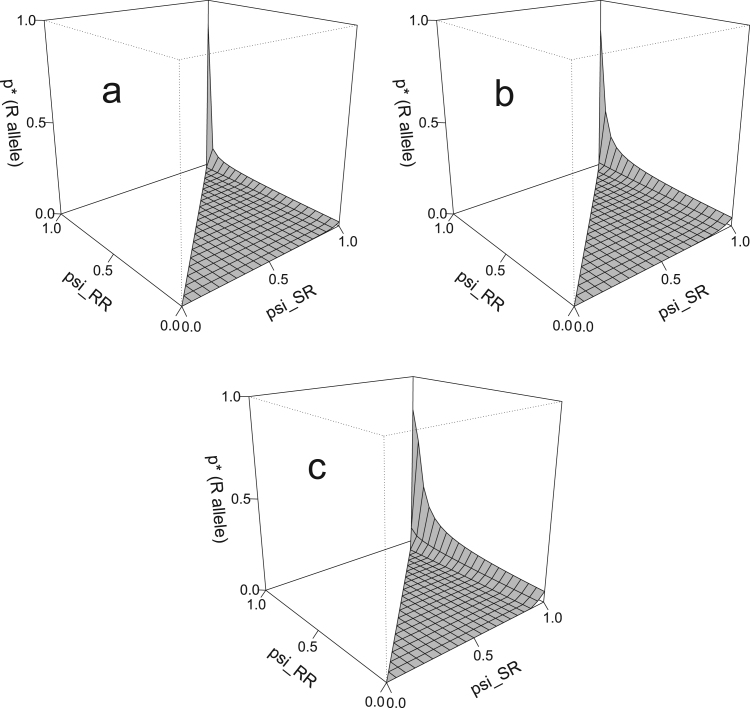
The effect of *ψ*_*SR*_ and *ψ*_*RR*_ on $p^{*}$ (the equilibrium *R* allele frequency) in the non-target deme. The model is spatial, with release ratio *d*=20, and a strong partially dominant incomplete resistance ($\gamma_{\mathrm{SR}}=0.2$, $\gamma_{\mathrm{RR}}=0.1$). Dispersal rates *m*=0.01 (a), *m*=0.05 (b) and *m*=0.1 (c) are used. Only points where $\psi_{\mathrm{SR}}\geq \psi_{\mathrm{RR}}$ (so that heterozygote resistance is always less costly than homozygote resistance) are shown. The threshold increase in $p^{*}$ can clearly be seen at high *ψ*_*SR*_ and *ψ*_*RR*_ values (low costs of resistance).

The *R* allele frequency $p^{*}$ in the non-target deme (Fig. 5) is much higher when there are very little or no fitness costs (as seen in the rear corner of all Fig. 5 panels). For most fitness cost combinations, the resistance goes extinct in the non-target deme ($p^{*}=0$), except where there are low or no fitness costs in *SR* heterozygotes (the right edge of all panels in Fig. 5). In this relatively small region of parameter space, the non-zero *R* allele frequency equilibria are slightly higher with higher rates of dispersal, while that region of parameter space itself is slightly larger. Only at, or very close to, zero costs does the *R* allele spread significantly in the non-target deme, with potentially detrimental effects on the pest control programme. Equilibrium plots of $N^{*}$ in the target deme (supplementary figure S6) show worse population suppression in regions where there are lower fitness costs of resistance, particularly in *SR* heterozygotes, with the worst degree of population control occurring where the *R* allele has no fitness costs. The impact of high resistance frequency in the non-target deme on the effectiveness of target population control is most clearly seen at higher dispersal rates due to the greater influx of *R* alleles from the non-target source.

### 3.3 Altering the effectiveness of resistance in a no-cost model

**Fig. 6:**
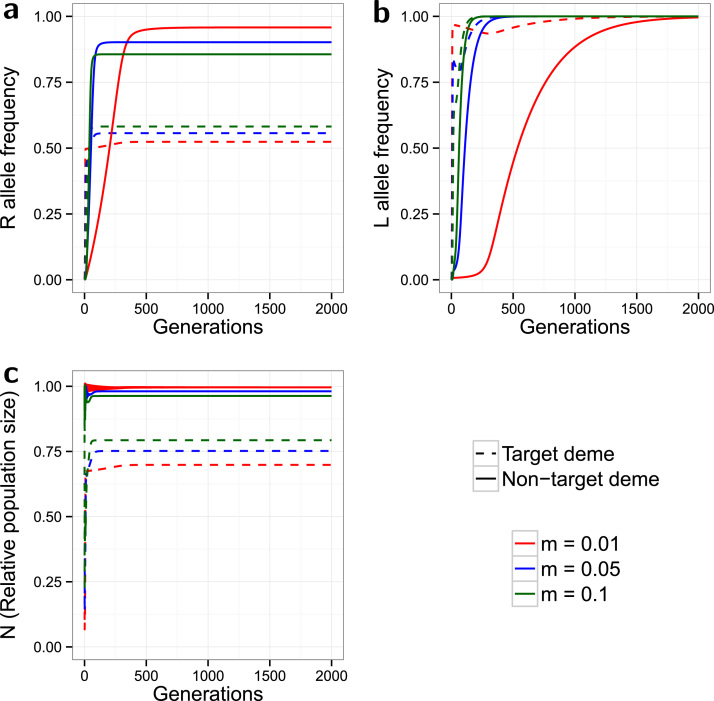
*Dominant, complete, no-cost resistance*. Evolution of the *R* allele frequency (a), the *L* allele frequency (b), and the change in the relative population size over time (c). The model is spatial, with release ratio *d*=20, and a dominant complete resistance ($\gamma_{\mathrm{SR}}=\gamma_{\mathrm{RR}}=0$) with no associated costs ($\psi_{\mathrm{SR}}=\psi_{\mathrm{RR}}=1$). Dashed lines indicate the target deme, solid lines indicate the non-target deme, and the line colours indicate the simulated dispersal rate (see legend). 2000 of the 3000 simulated generations are shown. The highly effective resistance spreads allows the lethal construct to reach fixation in both demes. Even in this worst-case scenario, some suppression of the target population is observed.

A dominant complete resistance with no associated costs (the strongest, most effective resistance possible) (Fig. 6) results in the *L* allele reaching near fixation in both demes for all values of *m*. The powerful resistance trait at higher frequencies reduces the selection against the *L* allele and allows it to spread to fixation. In the same manner as in the initial no-cost model (Fig. 2), the *R* allele equilibrium is higher in the non-target than in the target deme, and the deme equilibria are closer in value with a higher *m*. The non-target equilibria are also even higher than in Fig. 2, due to the greater selection for the more effective resistance, while target deme equilibria are very slightly lower. This latter result is most likely due to the reduced difference in population size between the two demes resulting in the target deme receiving a proportionately lower migratory influx of *R* alleles. The effect of *m* on population suppression is similar but the target deme *N* is higher overall due to the greater effectiveness of resistance.

**Fig. 7:**
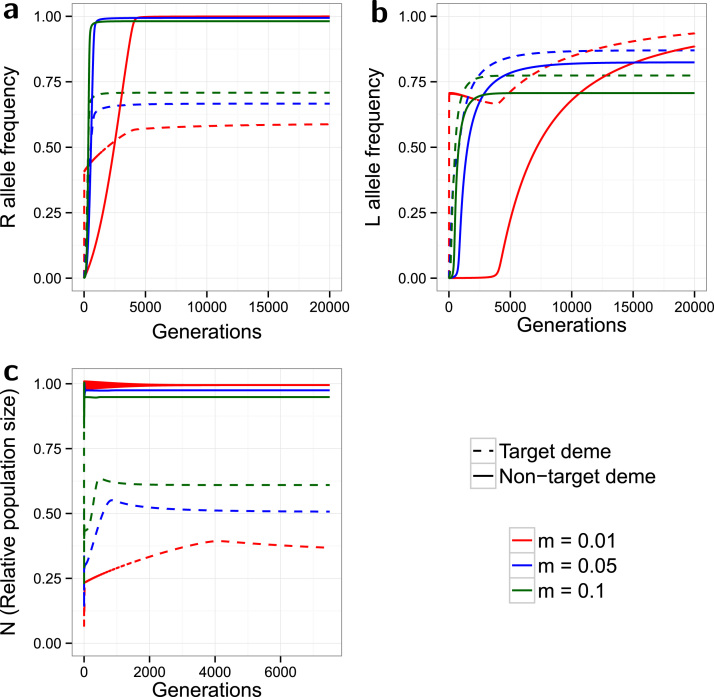
*Less dominant, complete, no-cost resistance*. Evolution of the *R* allele frequency (a), the *L* allele frequency (b), and the change in the relative population size over time (c). The model is spatial, with release ratio *d*=20, and a partially dominant complete resistance ($\gamma_{\mathrm{SR}}=0.4$, $\gamma_{\mathrm{RR}}=0$) with no associated costs ($\psi_{\mathrm{SR}}=\psi_{\mathrm{RR}}=1$). Dashed lines indicate the target deme, solid lines indicate the non-target deme, and the line colours indicate the simulated dispersal rate (see legend). Note the longer time scale (20,000 generations) than that shown in earlier figures. Only 7500 generations are shown for (c) to highlight the local peak in the target population size. The near fixation of resistance in the non-target deme drives an increase in the frequency of the lethal construct in that deme. Through migration, this increases the *L* allele frequency in the target deme also, which ultimately increases the effectiveness of target population control.

Increasing heterozygote resistance susceptibility ($\gamma_{\mathrm{SR}}=0.4$), while keeping homozygote susceptibility at 0, prevents the *L* allele reaching fixation in both demes for all *m*, although the $l^{*}$ values are still high (Fig. 7). Heterozygous resistance is no longer effective enough to counter the negative selection against the lethal construct, the spread of which is therefore limited. In this simulation it took very much longer to reach equilibria. This is a consequence of the weaker heterozygote resistance, as previously observed (Alphey et al., 2011). *R* allele equilibrium frequencies are higher in both demes (Fig. 7a) than they were with dominant complete resistance (Fig. 6a), with *R* nearing fixation in the non-target deme, particularly with a higher *m*. It is much more advantageous for an individual to have homozygous resistance, rather than heterozygosity, and therefore there is selection pressure for an increase in the *R* frequency even at high values of *p*. In the dominant complete model (Fig. 6) on the other hand, resistance is strong enough to provide good protection of the population from the genetic control even at lower *R* frequencies.

With all previously tested parameter combinations, higher dispersal brings the *L* allele frequencies in the two demes closer to each other. Uniquely, in the present case, *L* allele equilibria for the two demes are no longer closer together at higher *m* (so there is more going on than simply better mixing between the two populations), and instead higher dispersal causes lower $l^{*}$ in *both* target and non-target demes. In this sense the effect of dispersal rate on the non-target deme $l^{*}$ has been reversed. Observing the *L* curve for *m*=0.01 (Fig. 7b), we see that in the target deme *l* at first appears to be following earlier patterns (e.g. Fig. 2b) of declining towards a lower equilibrium point, but then the frequency suddenly starts increasing again from about the same time when the *R* allele in the non-target deme nears fixation. This suggests that the transient values of *l* in the target deme are initially consistent with previous patterns. However, once resistance has spread sufficiently far in the non-target deme that most individuals are homozygous resistant (and suffer no effects from the lethal construct), the negative selection against the *L* allele in this deme is drastically reduced, allowing a large increase in its frequency. Due to migration this increase in both *R* and *L* alleles is also exported to the target deme. The difference between the $p^{*}$ values in the two demes is caused by a balance between the dispersal rate *m* and the relative population sizes of the two demes.

Another qualitative difference in this set of simulations is that instead of rising to an equilibrium from the initial dip as seen previously, the target *N* (Fig. 7c) rises from this dip to a local peak before falling towards its equilibrium point. This change in the trajectory of *N* also appears to coincide with the rapid increase in *L* in this deme. The near fixation of resistance in the non-target deme drives an increase in the non-target *l* which, due to migration, in turn drives an increase in *L* alleles in the target deme. Because resistance is not fixed in that deme, this increases suppression of the target population. In this way the evolution of resistance in the non-target deme indirectly increases the effectiveness of population control in the target deme. Compared to the dominant complete model (Fig. 6), $N^{*}$ is lower for all values of *m* due to the above process and the overall lower effectiveness of resistance allowing greater population suppression. Further increasing the heterozygous susceptibility to achieve a co-dominant complete resistance ($\gamma_{\mathrm{SR}}=0.5$, $\gamma_{\mathrm{RR}}=0$) produces a more extreme pattern of results than seen in Fig. 7, even further away from those produced by the dominant complete model (Fig. 6) due to the lower effectiveness of heterozygous resistance.

### 3.4 Altering the susceptibility of resistance in a model with resistance costs

**Fig. 8:**
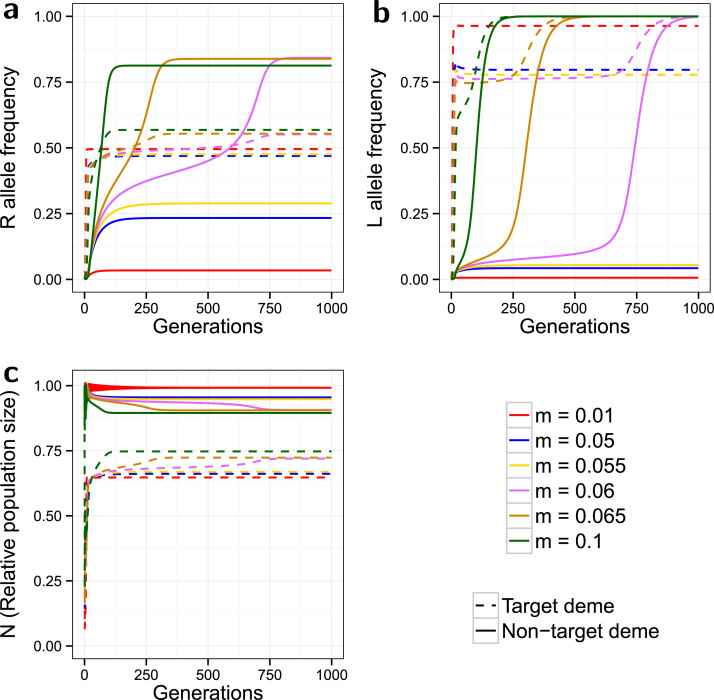
*Introducing minor costs of dominant, complete resistance*. Evolution of the *R* allele frequency (a), the *L* allele frequency (b), and the change in the relative population size over time (c). The model is spatial, with release ratio *d*=20, and a dominant complete resistance ($\gamma_{\mathrm{SR}}=\gamma_{\mathrm{RR}}=0$) with minor costs ($\psi_{\mathrm{SR}}=0.95$, $\psi_{\mathrm{RR}}=0.85$). Dashed lines indicate the target deme, solid lines indicate the non-target deme, and the line colours indicate the simulated dispersal rate (see legend). Greater influx of *L* alleles through dispersal into the non-target deme selects for a large increase in the frequency of resistance. This causes the non-target deme to become a source of *R* alleles for the target deme, which decreases the effectiveness of target population suppression.

A dominant complete resistance with minor fitness costs produces a very distinct and unexpected pattern (Fig. 8). For m<0.06, the *R* and *L* curves for both demes behave in a qualitatively similar manner to those in the original minor costs model with strong partially dominant incomplete resistance (Fig. 3). $p^{*}$ and $l^{*}$ in both demes are higher overall due to the more effective resistance and therefore population suppression is inferior. However the curves for m≥0.06 behave in a manner much more reminiscent of those for the no-cost model with dominant complete resistance (Fig. 6), with the non-target $p^{*}$ increasing above those of the target deme, and $l^{*}$ in both demes reaching fixation. For the *R* allele this *p* increase in the non-target deme coincides with a smaller increase in the target deme, while further increasing *m* still results in the equilibria in the two demes being closer in value.

This pattern is due to an increase in the dispersal rate increasing the influx of *L* alleles to the non-target deme enough that the (frequency-dependent) benefits of resistance there outweigh the fitness costs. Passing this threshold results in rapid proliferation of resistance in this deme, for those values of *m*, lowering the average fitness penalty of the *L* allele to the point that fixation can occur. It should be noted that the curves at higher *m* values are smooth for the non-target deme but seem to plateau before increasing again to equilibrium in the target deme. This further implies that it is the spread of resistance in the non-target deme that is driving these changes, with dispersal causing a concurrent increase in the target *p* and *l*, to the point that the latter also reaches fixation. It has already been established that increasing the costs of resistance lowers the selection for the *R* allele and limits its spread. This increase in resistance costs (compared to Fig. 6) therefore also limits the potential for the non-target deme to experience this upwards shifting behaviour in the *R* and *L* allele frequencies.

It is clear that target population suppression with the higher dispersal (*m* values) is inferior (higher $N^{*}$) compared with the lower *m* values, undoubtedly due to the higher levels of resistance that greater dispersal brings. This coincides with a lower non-target $N^{*}$ with the higher *m* values as consequence of net migration from the non-target to the target deme.

An overall very similar pattern is seen when the heterozygote susceptibility is slightly increased to give a partially dominant complete resistance ($\gamma_{\mathrm{SR}}=0.1$, $\gamma_{\mathrm{RR}}=0$) with the same minor fitness costs (supplementary figure S7). The difference here however is that the upwards shift in the *R* and *L* curves is no longer seen at *m*=0.06 but is seen at *m*=0.11. When resistance is less effective in heterozygotes, a higher dispersal rate is required to cause the critical influx of *L* alleles into the non-target deme required to initiate this upwards shift.

Increasing heterozygote susceptibility even further to give a co-dominant complete resistance ($\gamma_{\mathrm{SR}}=0.5$, $\gamma_{\mathrm{RR}}=0$) results in resistance and introgression of *L* alleles only emerging in the non-target deme, and only with the lowest dispersal rate *m*=0.01 (all other *R* and *L* curves remain at, or near, 0 frequency). In this case the dilution of resistance by the susceptible released GE insects is too strong for resistance to emerge, except in the non-target deme that has the lowest migratory influx of susceptibles. Large oscillations in the non-target *N*, reaching a stable limit cycle, with *m*=0.01 are again observed here, as seen in the model with costly resistance (Fig. 4). This pattern is almost identically reproduced when the homozygote susceptibility is increased to give a weak partially dominant incomplete resistance ($\gamma_{\mathrm{SR}}=0.5$, $\gamma_{\mathrm{RR}}=0.3$), thereby highlighting the primary importance of heterozygote resistance effectiveness.

## 4 Discussion

We have shown by mathematical simulation that incorporating spatial effects into a frequency-dependent population model of resistance to an engineered dominant lethal genetic construct, introduces a number of interesting and often counter-intuitive dynamic results.

We looked for (simulated) evidence of, and conditions for, dilution of resistance to the lethal construct in a population target for suppression, by susceptible insects from a nearby non-target population. This was in anticipation of parallels with the dilution of resistance to engineered insecticidal (Bt) crops by susceptible insects from refuges of non-transgenic plants (Alphey et al., 2007, Tabashnik et al., 2009). If overall selection for resistance is low enough then we did indeed observe that the non-target population acts as a source of susceptible alleles for the target population, thereby limiting the spread of resistance in this latter population. This effect arises from a combination of higher fitness costs and/or a low effectiveness of resistance. The dilution of resistance restricts the adverse effect of such a resistant allele on the degree of control (suppression or local elimination) of the target population that may be achieved. However, whether this population control is more or less effective than in a non-spatial model, representing an isolated population, depends on the magnitude of migration from the relatively unsuppressed non-target population.

Conversely, a key finding from our model is the potential for a high selective pressure for resistance to drive a very high equilibrium frequency of resistance in the non-target population, where resistance dilution from the homozygous susceptible males is limited. A critical strength of net positive selection can be achieved through a combination of low costs and a high effectiveness of resistance. In these cases the non-target population becomes a source of resistant alleles and increases the frequency of resistance in the target population also, particularly with higher dispersal rates between demes. This in turn reduces the effectiveness of population control.

It is not simply the case that the resistant allele's fitness properties assign the non-target deme irrevocably and unequivocally to be either a source of *R* alleles or a source of *S* alleles and thus seal the fate of a planned programme of genetic population control. Interesting dynamic behaviours can emerge from the interplay of genetic traits and spatial dynamics, with dispersal both into and out of the target population playing a role over time. For example, where (partially dominant) resistance is complete in homozygotes, but incomplete in heterozygotes, the dispersal of the genetic construct away from the release site, and low fitness costs of resistance, can allow a very high equilibrium frequency of resistance to emerge in the non-target population. As resistance reaches near fixation, a critical population protection threshold is crossed which drives a rapid increase in the frequency of the lethal construct in this deme. Migration then drives a rapid increase in the frequency of the lethal construct in the target population, which ultimately results in stronger suppression of the target population, a favourable outcome from a programme manager's perspective. This process implies that spatial effects could make an insect population with a partially dominant and complete resistance relatively amenable to genetic control (as long as the non-target population is not also of economic or public health importance).

The dynamics observed in this spatial model could have different implications for the use of genetic control depending on the type of control being implemented. If the release strategy is static, meaning that it is only ever applied in a single target population, then many of the processes described in these results could come into play. For example, if a very effective resistance with low costs emerges, then this resistance could proliferate in the (non-target) populations in the surrounding area, and then indirectly decrease the efficacy of genetic control in the target population through migration. This process may not be detectable without detailed population genetic monitoring of resistance frequencies in both the target and the non-target populations. A rolling control programme, on the other hand, where modified insect releases are first applied to a target population before being extended to surrounding populations, raises additional questions. For instance, if a high frequency of resistance evolves in a non-target population, could this evolution be slowed or reversed by resistance dilution once genetic control is extended and applied directly to this population? Resistance in any of these scenarios could also be combated by switching to alternative engineered strains which are not affected by the field-evolved resistance (i.e. no cross-resistance).

In a related concurrent study, Thompson (2015) used a constant-number release policy and absolute population sizes in a non-spatial model, finding qualitatively similar results to the proportional release model (Alphey et al., 2011). This constant release assumption, arguably closer to practical reality, merits further investigation in a spatial model. However with the insights provided by our model, the qualitative explanations for our findings are still expected to hold true (albeit at different values and thresholds). For example, no-cost, modestly effective resistance has a major advantage in the non-target deme against the lethal construct, which leaks in from the target deme, and has no selective disadvantage. Resistance therefore spreads extensively in the non-target deme, which then acts as a source of *R* alleles to the target deme. Very high-cost resistance on the other hand will go extinct in both demes. It is reasonable to anticipate that both of these insights would remain true in a constant-release model, although how ‘modestly effective’, and how ‘high’ the costs of, resistance must be to fall within that region of outcome, would most likely be different under the two assumptions.

We do not simulate dynamics after cessation of releases, where the only benefit of resistance would be against legacy *L* alleles. Unless the *R* allele is fixed (unlikely in the target deme, which was flooded with released susceptible insects), resistance with associated fitness costs should fade away, and at a faster rate with greater costs. The *L* allele should also die out, unless there is complete resistance remaining in the population. Experiments with the olive fruit fly *Bactrocera oleae* and diamondback moth *Plutella xylostella*, bearing female-lethal transgenic constructs, showed that the construct frequency fell by approximately 50% each generation (Harvey-Samuel et al., 2014). This is as expected due to the construct killing a half of its carriers, with female offspring dying but males surviving. The decrease in frequency could also potentially be faster if the construct had significant fitness costs in males. Transgenes at an initial frequency of 0.25 in populations of 200 insects went extinct in 11 generations or fewer, however the presence of a resistant allele would be expected to slow this decay. Given the sometimes non-intuitive nature of the spatial effects in our model, it is difficult to make predictions about the relative rates of allele extinction in the two demes.

When judging the relative effectiveness of genetic control in these results it is important to consider what level of population suppression is actually required to meet the goals of the programme. In all our simulations, the target equilibrium population size is not as low as that which would be achieved if no resistance were present. However, population suppression might still be sufficient to limit damage from an agricultural crop pest below the threshold for economic harm. Similarly, for pests that act as vectors for disease, the population could potentially still be suppressed below the entomological threshold to sustain disease transmission. While the scenarios presented here that result in superior population suppression are more likely to meet these criteria, additional studies, that include the use of economic and epidemiological models, would be required for formal predictions to be made.

Stable oscillations in the population size of the non-target deme, with a low dispersal rate, were observed in some simulations (costly partially dominant incomplete resistance, Fig. 4; or co-dominant complete resistance). This phenomenon is a consequence of the formula for density dependence used in this model creating a bifurcation surface for population size under certain conditions. Bifurcations of this kind are typically produced by changing the effective rate of reproductive increase and/or the strength of the density dependent feedback (Hassell et al., 1976). The population growth parameter (*R*_0_) is kept constant in this model, but the effective population growth rate is also influenced by the effective ratio of fertile to ‘genetically sterile’ males, which reduces reproduction by reducing the fraction of matings that are successful. That fraction is modified by the extent to which resistance protects individuals from inherited lethal genes. The survival advantage and the associated fitness penalty (particularly if very costly) of a resistant allele, thus have the potential to alter the strength of the density dependent feedback, especially in the non-target deme which has a higher population size. Dispersal also has the potential to produce complex dynamics, although such effects depend on the model construct (Sinha and Parthasarathy, 1994, Dey et al., 2014); the Bellows model has been shown to exhibit periodic dynamics under emigration (Agarwal and Sinha, 2005). Additional study of the specific mathematical processes involved (i.e. stability analysis) would be needed to shed further light on this topic and whether it is of mathematical and/or ecological significance.

We have not explicitly presented simulations for a few alternative combinations of homozygous and heterozygous resistance effectiveness (e.g. recessive complete resistance, $\gamma_{\mathrm{SR}}=1$, $\gamma_{\mathrm{RR}}=0$). However, it is clear that the parameter combinations used in this study are sufficient for determining the general impact of the effectiveness of resistance and the relative importance of homozygous vs. heterozygous effectiveness. The effectiveness of heterozygous resistance is the major driver behind the observed dynamics, in accordance with our previous non-spatial findings (Alphey et al., 2011). In some circumstances the homozygous effectiveness has been shown also to play an important role (Fig. 7).

The type of genetic construct investigated here (bisex and late acting lethality) is applicable to GE strains such as OX513A *A. aegypti*, which targets the main mosquito vector of dengue fever (Phuc et al., 2007), but could be altered in further studies to tailor results for alternative strains and target species. Our non-spatial model (Alphey et al., 2011) found qualitatively similar results from simulations with bisex and female-specific lethality, although this might not necessarily be the case in a spatial model.

The point in the insect life cycle at which dispersal predominantly occurs could differ in some species. If dispersal were modelled to occur before mating instead, there could be a large influx of released GE males into the non-target population also. However the assumption in our model, of limited dispersal occurring after mating, is applicable to a number of key target insect species. For example, diamondback moths mate at dusk on the day they emerge (Talekar and Shelton, 1993). The vast majority of males and females only make short trivial flights, remaining on their natal crops, although a small proportion (<1%) travel further and long-distance seasonal migrations have been observed (Furlong et al., 2013). For *A. aegypti* mosquitoes, closely adjoining neighbourhoods would be part of a single population (requiring an area-wide release programme across that whole district), but male mosquitoes do not disperse across open spaces, or travel far at all, unless necessary to find resources such as food and mates (Harrington et al., 2005, Hemme et al., 2010, Trpis et al., 1995). In defining the landscape scale, and planning the releases, we may therefore reasonably assume that two distinct populations are connected by only a small fraction of adults at the high end of the dispersal scale distribution.

The use of continuous state variables in our deterministic model means that complete fixation or loss of an allele cannot occur mathematically. Extending this model to include stochastic dynamics would therefore be a useful development and might reveal any important differences. For example, if the initial suppression of a population is good enough, potentially the population could be eliminated though stochastic effects before resistance emerges and limits the effectiveness of control. Similarly, at very low frequencies the resistant (*R*) or susceptible (*S*) alleles, or the wild type (*w*) absence of transgene at the locus of the lethal construct, could be lost from the population, while the relevant corresponding allele would reach fixation. In a spatial model, an allele would have to be lost, or a population would have to be eradicated, from both demes in order to disappear entirely, as migration will quickly reintroduce alleles and individuals. Stochasticity could also have a major impact on dynamics where critical thresholds exist that can drive alternative outcomes.

We assume a single resistant gene in the population, with constant trait values for the effectiveness of resistance and its fitness costs. A further interesting study could start with a high cost and relatively ineffective resistance, and explore the potential evolution of fitness modifiers by modelling a number of resistant alleles with different values, and allowing mutation between types. This could model the time dynamics of evolutionary change in the resistant allele simultaneously with the population dynamics of the genetic control method. This might be done, for example, by adapting evolutionary ecology methods that apply Price's equation to model evolutionary change in a pathogen population coupled with epidemiological dynamics (Day and Gandon, 2006).

Further development of this model could focus on varying the spatial population structure and patterns of dispersal. In reality a target population may be surrounded by a larger non-target population or group of populations, a scenario that could be simulated by simply increasing the *N*_0_ of the non-target deme (or by expanding to a network model). The study of source/sink population dynamics, or a directional bias in migration, between the two demes could also prove fruitful and would increase the relevance of this model for a wider variety of ecological settings. Furthermore, the flight potential of wild type and transgenic insects can differ (Bargielowski et al., 2012), a scenario that could be modelled by using different dispersal rates for these two groups of genotypes when calculating migration between the demes. Rather than a constant proportion of a population dispersing to another deme, the departure of insects could be density dependent. This is a promising area for further study, as there are a large number of additional factors that could have a significant effect on the spatial evolution of resistance.

Pest insects are notorious for developing resistance, physiological or behavioural, to diverse forms of pest management. Genetics-based methods, like any other effective control, impose strong selection pressure in favour of any allele that confers resistance to the control mechanism. The built-in resistance dilution (through releasing susceptible males) may lessen the risk somewhat, by presenting an evolutionary barrier that weak unfit resistant alleles are unable to surpass. However, it is still likely that the self-limiting genetic strategy modelled here would be implemented as part of an integrated pest management or integrated vector control programme. Deployment of multiple pest management tools with independent modes of action, for example engineered male releases with biopesticides such as Bti, serves to slow the evolution of resistance to any one of the components.

In conclusion, we have highlighted the importance of spatial effects in the evolution of resistance to a self-limiting genetic insect control method based on the sterile insect technique. Depending on the nature of the resistance and the rate of dispersal, spatial dynamics can drive an increased or decreased evolution of resistance in both the target and non-target population, compared with that predicted for otherwise identical resistance in an isolated target pest population. This evolution could have a significant impact, positive or negative, on the effectiveness of genetic population suppression, through a variety of interacting population genetic and population dynamic processes. These considerations could influence priorities for research and development, for example, which species to prioritise for product development, and how much effort to devote to measuring dispersal behaviour in field settings. Our findings also have practical implications for field trials and implementation programmes. For example, the number and placement of traps in and outside of the focal area for entomological surveillance, and knowledge of how changes in nearby populations (e.g. the stability of population dynamics in the field, or performance of wild-caught insects in laboratory tests for resistance) might presage evolutionary effects in the population that has been targeted for control. Spatial interactions between a target population and insects in a non-target area can have a range of possible outcomes, from providing extra dilution to further slow or even prevent the spread of resistance in the target population, thus improving the efficacy of the control programme, to at the other extreme exacerbating the evolution of resistance.

## Figures and Tables

**Table 1:** Parameters and variables used in the mathematical model.

**Symbol**	**Parameter/variable description**	**Constraints/values**
*p*	Frequency of resistant *R* allele in current adult generation	0≤p≤1
*q*	Frequency of susceptible *S* allele	0≤q≤1,p+q=1
*p*_0_	Initial *R* allele frequency	0.001
$p^{*}$	Equilibrium *R* allele frequency	
*l*	Frequency of the (GE) lethal genetic construct *L*	0≤l≤1
*i*	Genotype at S/R locus	*SS*, *SR* or *RR*
*j*	Genotype at L/w locus (*w* is the wild-type absence of the lethal construct)	*LL*, *Lw* or *ww*
*ψ*_*i*_	Relative fitness of larvae of genotype *i* (costs of resistance)	$0\leq \psi_{\mathrm{RR}}\leq \psi_{\mathrm{SR}}\leq \psi_{\mathrm{SS}}=1$
*ϵ*_*k*_	Fitness penalty of lethal construct (to both sexes)	$\epsilon_{k}=1$
*γ*_*i*_	Susceptibility to the lethal construct (scaling factor applied to fitness penalty *ϵ*)	$0\leq \gamma_{\mathrm{RR}}\leq \gamma_{\mathrm{SR}}\leq \gamma_{\mathrm{SS}}=1$, $\gamma_{\mathrm{RR}}\neq \gamma_{\mathrm{SS}}$
*η*_*j*_	Number of copies of the lethal construct	0 for *ww*, 1 for *Lw*, or 2 for *LL*
*Ω*_*ijk*_	Relative fitness of larvae of genotype i,j,k	$0\leq \Omega_{\mathrm{ijk}}\leq 1$
*d*	Release ratio of GE males to the total number of males in the wild population at that generation	1, 20, or 50
*R*_0_	Average number of female progeny produced per adult female in its lifetime (a single generation)	7.5
*N*_*t*_	Population size of mature adults at generation *t* relative to the initial population size	$N_{0}=1$
*F*_*t*_	Relative population size of mature females at generation *t*	*F*_0_=0.5
F^t	Migration adjusted female population size	
*σ*_*t*_	Simulated proportion of all offspring that survive to maturity	
*m*	Dispersal rate between demes (the proportion of the resident population emigrating)	0≤m≤1
*Z*_*t*_	Matrix of zygote genotype frequencies in generation *t*	

**Table 2:** Designated parameter values for the resistance trait. $\gamma_{\mathrm{SS}}=\psi_{\mathrm{SS}}=1$ in all simulations. Relative fitnesses for each combination of parameter values by genotype are detailed in supplementary table S1.

**Parameter designation**	**Constraints/values**
Dominant complete	$\gamma_{\mathrm{SR}}=\gamma_{\mathrm{RR}}=0$
Partially dominant complete	$1>\gamma_{\mathrm{SR}}>0$, $\gamma_{\mathrm{RR}}=0$
Co-dominant complete	$\gamma_{\mathrm{SR}}=0.5$, $\gamma_{\mathrm{RR}}=0$
Partially dominant incomplete	$1>\gamma_{\mathrm{SR}}>\gamma_{\mathrm{RR}}>0$
No costs	$\psi_{\mathrm{SR}}=\psi_{\mathrm{RR}}=1$
Minor costs	$\psi_{\mathrm{SR}}=0.95$, $\psi_{\mathrm{RR}}=0.85$
Fit resistance	$\psi_{\mathrm{SR}}=0.9$, $\psi_{\mathrm{RR}}=0.7$
Costly resistance	$\psi_{\mathrm{SR}}=0.2$, $\psi_{\mathrm{RR}}=0.1$

## Appendix A Supplementary data

Application 1
