# Supplementary material for: Resistance to genetic insect control: Modelling the effects of space
Source: J Theor Biol. 2017 Jan 21;413:72–85. doi: 10.1016/j.jtbi.2016.10.014 (PMC5177727; doi:10.1016/j.jtbi.2016.10.014)
Supplement: Application 1 [file mmc1.pdf]

Supplementary Figures and Results

Resistance to genetic insect control: Modelling the effects of space Supplementary Material

Watkinson-Powell, Benjamin

and

Alphey, Nina

1 Introduction

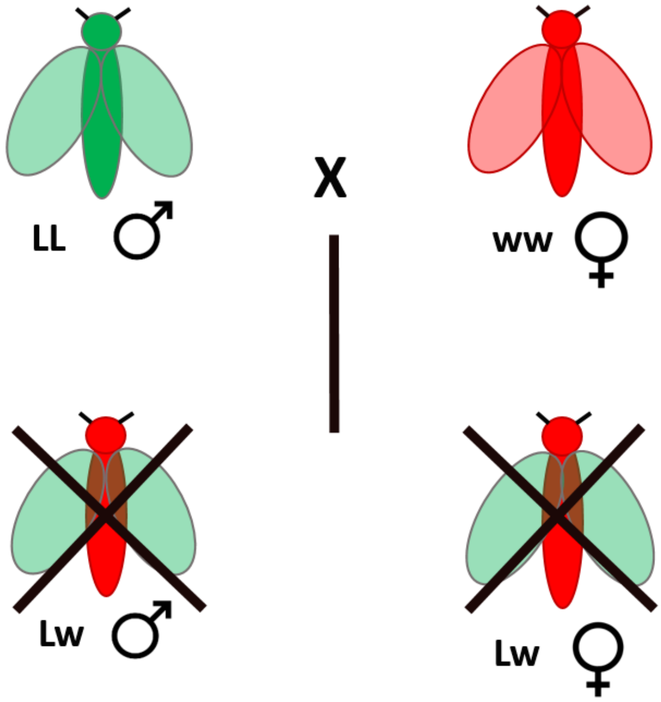

Figure S1: The inheritance pattern of the lethal construct. GE males (LL) mate with wild-type females (ww), and the construct is inherited by F_1 progeny, causing death in both sexes.

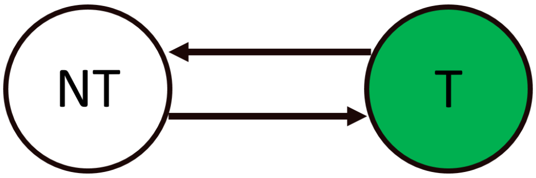

Figure S2: Representation of the two deme spatial structure of the mathematical model. GE males (green) are released into the target deme (T), which is connected to the non-target deme (NT) by dispersal.

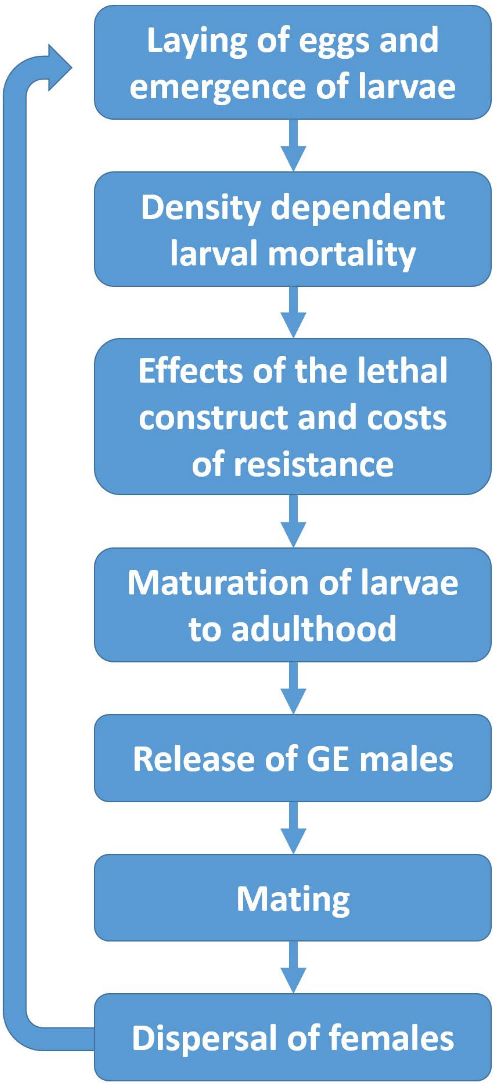

Figure S3: Representation of the insect life cycle stages used in the mathematical model. The returning arrow represents the start of a new insect generation.

2 Methods

Table S1: Fitnesses for each combination of resistance parameter values by genotype.

Genotype:	SSww	SRww	RRww	SSLw	SRLw	RRLw	SSLL	SRLL	RRLL
**dominant complete resistance**									
no costs	1	1	1	0	1	1	0	1	1
minor costs	1	0.95	0.85	0	0.95	0.85	0	0.95	0.85
fit resistance	1	0.9	0.7	0	0.9	0.7	0	0.9	0.7
costly resistance	1	0.2	0.1	0	0.2	0.1	0	0.2	0.1
**partially dominant complete** $(0 < \gamma_{SR} < 1)$									
no costs	1	1	1	0	1-γ_{SR}	1	0	$(1-\gamma_{SR})^2$	1
minor costs	1	0.95	0.85	0	$0.95(1-\gamma_{SR})$	0.85	0	$0.95(1-\gamma_{SR})^2$	0.85
fit resistance	1	0.9	0.7	0	$0.9(1-\gamma_{SR})$	0.7	0	$0.9(1-\gamma_{SR})^2$	0.7
costly resistance	1	0.2	0.1	0	$0.2(1-\gamma_{SR})$	0.1	0	$0.2(1-\gamma_{SR})^2$	0.1
**co-dominant complete**									
no costs	1	1	1	0	0.5	1	0	0.25	1
minor costs	1	0.95	0.85	0	0.475	0.85	0	0.2375	0.85
fit resistance	1	0.9	0.7	0	0.45	0.7	0	0.225	0.7
costly resistance	1	0.2	0.1	0	0.1	0.1	0	0.05	0.1
**partially dominant incomplete** $(0 < \gamma_{RR} < \gamma_{SR} < 1)$									
no costs	1	1	1	0	1-γ_{SR}	1-γ_{RR}	0	$(1-\gamma_{SR})^2$	$(1-\gamma_{RR})^2$
minor costs	1	0.95	0.85	0	$0.95(1-\gamma_{SR})$	$0.85(1-\gamma_{RR})$	0	$0.95(1-\gamma_{SR})^2$	$0.85(1-\gamma_{RR})^2$
fit resistance	1	0.9	0.7	0	$0.9(1-\gamma_{SR})$	$0.7(1-\gamma_{RR})$	0	$0.9(1-\gamma_{SR})^2$	$0.7(1-\gamma_{RR})^2$
costly resistance	1	0.2	0.1	0	$0.2(1-\gamma_{SR})$	$0.1(1-\gamma_{RR})$	0	$0.2(1-\gamma_{SR})^2$	$0.1(1-\gamma_{RR})^2$

3 Results

3.1 Spatial vs. non-spatial for a no-cost resistance

For the initial spatial model alternative release ratios (d) were also tested. With $d = 1$ (figure S4) the results show a lower l^*, higher p^* and a higher N^* in the target deme, compared to $d = 20$ (figure 2), for all values of m. The release of fewer GE insects means that the influx of L alleles into the target deme, and therefore the suppressive impact on population size, is lower, but also that resistance dilution by the released susceptible males is reduced. l^* in the non-target deme is slightly higher whereas almost no change in the value of p^* or N^* is seen. This increase in the non-target l^* is explained by the higher N^* in the target deme producing greater total migration, and therefore a greater influx of L alleles. All equilibria are reached faster with this lower value of d, potentially due to the lower susceptible dilution allowing resistance to spread faster. Transient oscillations can be seen in the non-target N, due to the genetic construct's perturbation of the coupled system, however these converge to stable equilibrium values. The results from $d = 50$ (not shown) on the other hand are almost identical to those with $d = 20$ (figure 2) with the exception that equilibria take slightly longer to be reached. It must be assumed that GE control reaches near maximum efficacy at or below $d = 20$ and therefore any higher release ratio is redundant.

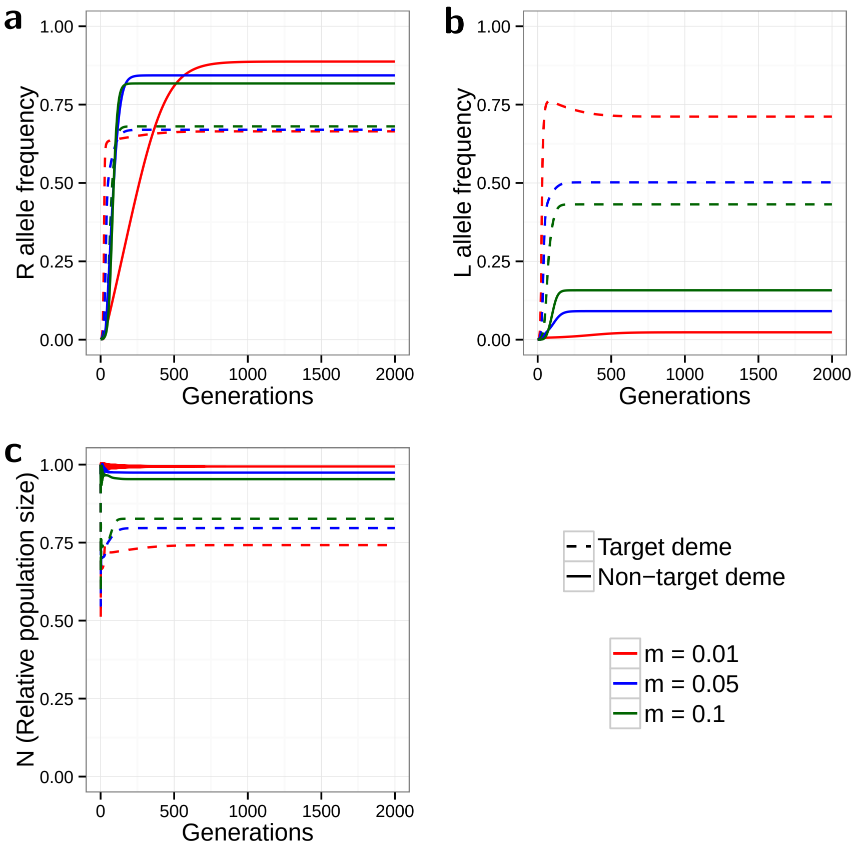

Figure S4: A lower release ratio, with no-cost resistance - Evolution of the R allele frequency (a), the L allele frequency (b), and the change in the relative population size over time (c). The model is spatial, with release ratio $d = 1$, and a strong partially dominant incomplete resistance ($\gamma_{SR} = 0.2$, $\gamma_{RR} = 0.1$) with no associated costs ($\psi_{SR} = \psi_{RR} = 1$). The parameter values and starting conditions are identical to Figure 2, except for the release ratio. Dashed lines indicate the target deme, solid lines indicate the non-target deme, and the line colours indicate the simulated dispersal rate (see legend). 2000 of the 3000 simulated generations are shown. These results, and those for $d = 50$ (not illustrated), both indicate qualitatively similar behaviours to Figure 2. Here, the lower release ratio results in less effective population control, due to a reduced spread of the lethal construct and a lessened dilution of resistance.

3.2 Adding costs of resistance

The model with intermediate cost 'fit resistance' (figure S5) produces qualitatively similar results to the minor costs model (figure 3). The only major deviation seen here in this model is that the R allele frequency in the non-target deme does not increase much above p_0, as is seen for the L allele in both this and figure 3. The higher costs of resistance reduce the positive selection for resistance and prevent it from spreading in this deme. Furthermore the decrease in the target p^* and l^* with increasing m is greater than in the minor costs model, to the point that the R and L alleles do not emerge with $m = 0.1$. N^* in the target deme is slightly lower than in the minor costs model

(improved population suppression due to the lower resistance frequency), with a greater decrease being seen for $m = 0.1$.

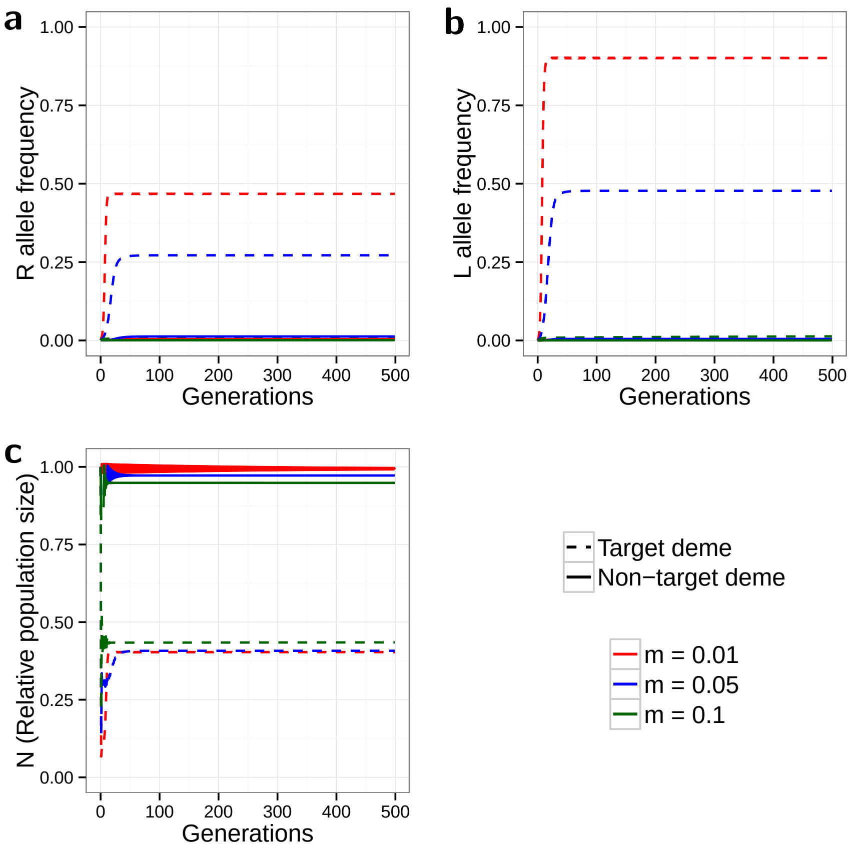

Figure S5: Intermediate costs of resistance - Evolution of the R allele frequency (a), the L allele frequency (b), and the change in the relative population size over time (c). The model is spatial, with release ratio $d = 20$, a strong partially dominant incomplete resistance ($\gamma_{SR} = 0.2$, $\gamma_{RR} = 0.1$), and fit resistance ($\psi_{SR} = 0.9$, $\psi_{RR} = 0.7$). Dashed lines indicate the target deme, solid lines indicate the non-target deme, and the line colours indicate the simulated dispersal rate (see legend). The costs of resistance prevent it from spreading in the non-target deme, and limit its spread in the target deme, with the result that target population suppression is improved (compared to figure 3).

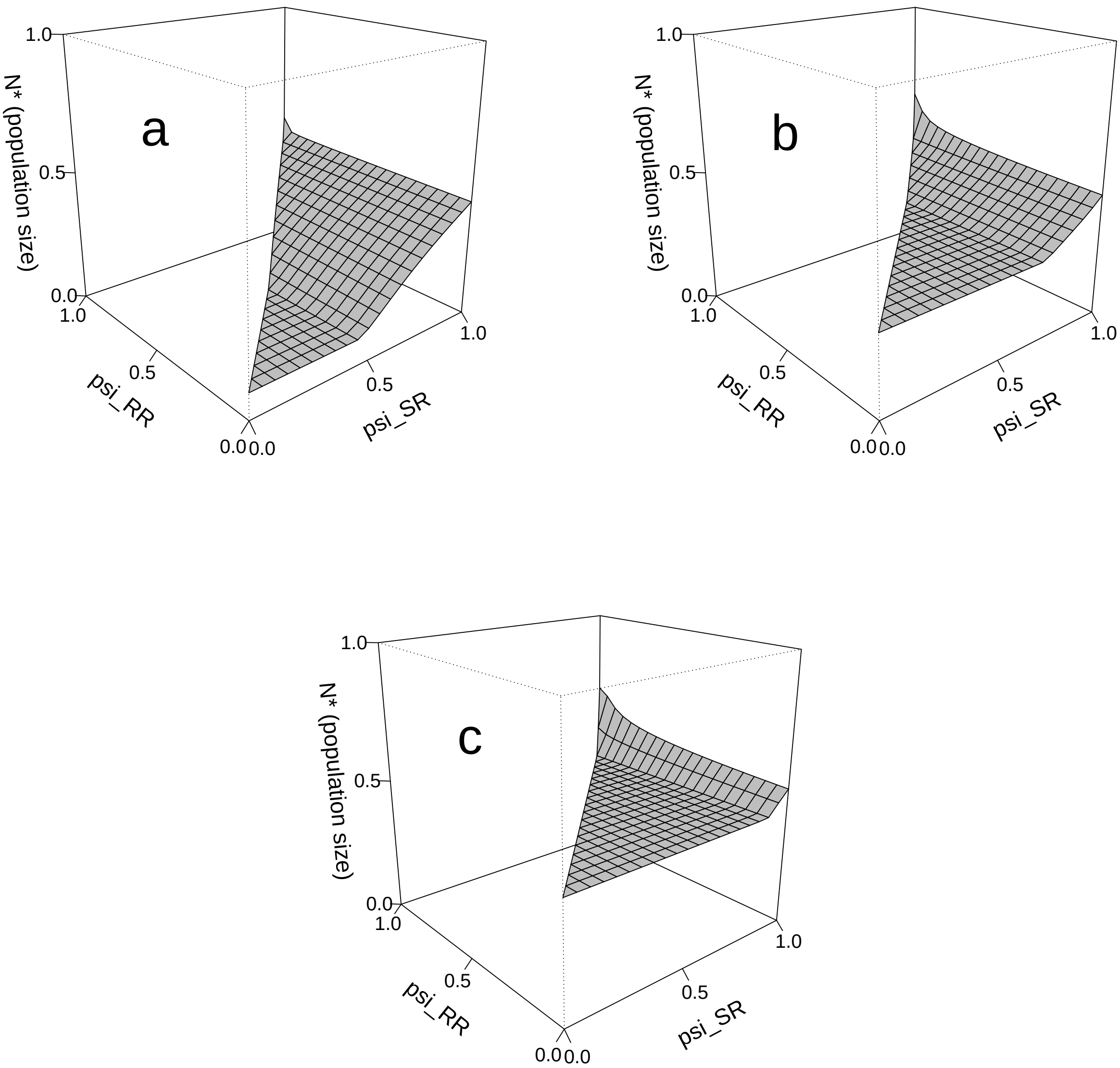

Figure S6: The effect of ψ_{SR} and ψ_{RR} on N^* (the equilibrium population size) in the target deme. The model is spatial, with release ratio $d = 20$, and a strong partially dominant incomplete resistance ($\gamma_{SR} = 0.2$, $\gamma_{RR} = 0.1$). Dispersal rates $m = 0.01$ (a), $m = 0.05$ (b) and $m = 0.1$ (c) are used. Only points where $\psi_{SR} \geq \psi_{RR}$ (so that heterozygote resistance is always less costly than homozygote resistance) are shown. The effect of the threshold increase in the non-target deme p^* at high ψ_{SR} and ψ_{RR} values (low costs of resistance), on the target deme N^*, is most apparent at higher dispersal rates. In these cases the non-target deme acts as a source of R alleles and reduces the effectiveness of population control in the target deme.

3.3 Altering the susceptibility of resistance in a model with resistance costs

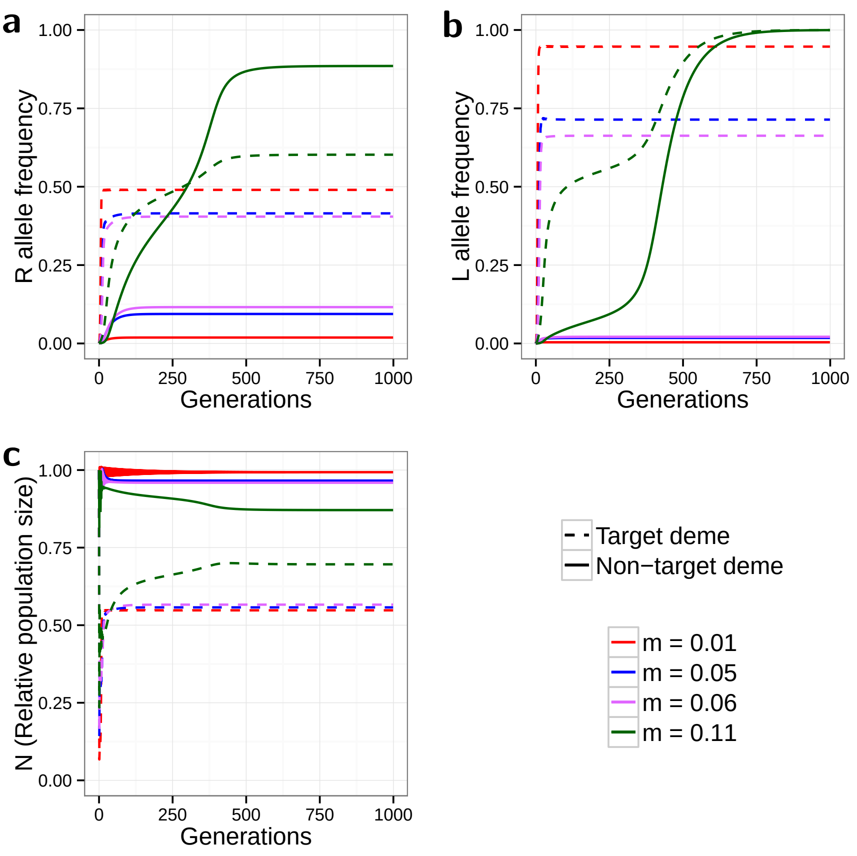

Figure S7: Incomplete heterozygote resistance with minor costs - Evolution of the R allele frequency (a), the L allele frequency (b), and the change in the relative population size over time (c). The model is spatial, with release ratio $d = 20$, and a partially dominant complete resistance ($\gamma_{SR} = 0.1$, $\gamma_{RR} = 0$) with minor costs ($\psi_{SR} = 0.95$, $\psi_{RR} = 0.85$). Dashed lines indicate the target deme, solid lines indicate the non-target deme, and the line colours indicate the simulated dispersal rate (see legend). Less dominant resistance means that a higher dispersal rate is required (compared to figure 8) for the influx of L alleles in the non-target deme to drive a threshold increase in the R allele frequency.
